# Cost-benefit analysis of implementing Vendor Neutral Archive (VNA) technology in the Brazilian Public Health System

**DOI:** 10.31744/einstein_journal/2025GS1219

**Published:** 2025-08-08

**Authors:** Giulia Osório Santana, Luis Gustavo Nascimento de Paula, Leandro Seiti Anazawa, Pedro Vieira Santana Netto, Aline de Souza Moura, Daniel Tornieri, Joselisa Péres Queiroz de Paiva, Rafael Maffei Loureiro, William Yang Chen Fan, Ligia Bueno Sandes, Lucas Reis Correia

**Affiliations:** 1 Hospital Israelita Albert Einstein São Paulo SP Brazil Hospital Israelita Albert Einstein, São Paulo, SP, Brazil.; 2 Department of Preventive Medicine Faculdade de Medicina Universidade de São Paulo São Paulo SP Brazil Department of Preventive Medicine, Faculdade de Medicina, Universidade de São Paulo, São Paulo, SP, Brazil.

**Keywords:** Cost-benefit analysis, Diagnostic imaging, Cloud computing, Vendor neutral archive, Government programs, Public Health, Information storage and retrieval, System integration, Radiology Information Systems, Brazil

## Abstract

**Objective:**

To conduct an *ex-ante* cost-benefit analysis of implementing Vendor Neutral Archive technology in the Brazilian Public Health System.

**Methods:**

The cost-benefit analysis involves monetizing the potential costs and benefits of implementing Vendor Neutral Archive in the Brazilian Public Health System, focusing on comparing these values using the return on investment measure.

**Results:**

The aggregate analysis at the Brazilian Public Health System level reveals that the estimated return on investment for the first year after Vendor Neutral Archive implementation is 5.63%, rising to 10.12% after three years. The results indicate a positive correlation between the number of imaging exams conducted and the financial gains for the health institution under analysis. A minimum number of approximately 11,500 exams is required to implement Vendor Neutral Archive technology to generate positive returns in the first year after implementation.

**Conclusion:**

Despite the importance of interpreting results cautiously owing to assumptions and methodological limitations, the findings highlight the strategic significance of Vendor Neutral Archive in enhancing the efficiency and sustainability of the Brazilian Public Health System. Adopting cloud-based medical image storage requires a significant upfront investment but promises substantial benefits. Within the Brazilian Public Health System, the return on this investment can be realized in less than one year, correlated with the volume of imaging exams performed. This underscores the importance of scalability in the healthcare system, where wider adoption of Vendor Neutral Archive results in higher returns.

## Highlights

■ Vendor Neutral Archive implementation has an return on investment of 5.63% in the first year and 10.12% after three years.

■ At least 11,500 exams are needed annually for a positive return on investment in the first year after implementation.

■ Vendor Neutral Archive enhances interoperability and efficiency by standardizing and centralizing image storage.

■ Scalability is key: higher adoption of Vendor Neutral Archive leads to greater financial and operational benefits.

## In Brief

Vendor Neutral Archive is a centralized, cloud-based solution that enables interoperable storage and management of medical images. Vendor Neutral Archive enhances data accessibility and long-term cost efficiency, supporting a more scalable and sustainable healthcare infrastructure. This study conducts a cost-benefit analysis of implementing Vendor Neutral Archive technology in the Brazilian Public Health System.

## Graphical abstract

**Figure:**
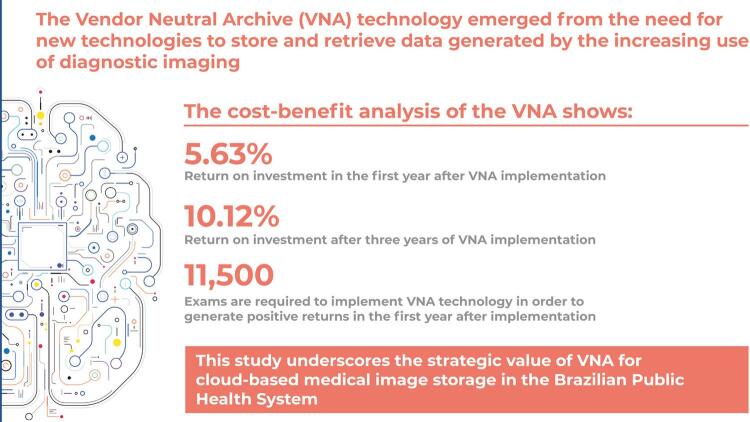

## INTRODUCTION

Diagnostic imaging enables medical professionals to analyze patient health conditions earlier, more accurately, and less invasively, leading to more appropriate treatments. The growth of this field is driven by advancements in imaging technologies, as evidenced by the expanded use of radiography, ultrasonography, computed tomography, magnetic resonance imaging, and other modalities. This underscores the crucial role of developing these tools in the medical field.^(1)^

The increasing use of diagnostic imaging has necessitated new technologies to store and retrieve the resulting data. To address this, the *Hospital Israelita Albert Einstein* (HIAE) launched the *Banco de Imagens* project under the *Programa de Apoio ao Desenvolvimento Institucional do Sistema Único de Saúde* (PROADI-SUS). This project aims to create a national database of medical images using Vendor Neutral Archive (VNA) technology.

Vendor Neutral Archive technology is characterized by its neutrality of image type, allowing long-term storage and processing using standardized formats and interfaces. Its interoperability, scalability, and data protection capabilities facilitate efficient integration between information systems from different suppliers,^(2-4)^facilitating more efficient transmission of exam data from diagnostic imaging centers (DICs) to medical facilities.

### OBJECTIVE

This study aims to perform an *ex-ante* cost-benefit analysis of the development and implementation of Vendor Neutral Archive in the Brazilian Public Health System.

## METHODS

The cost-benefit analysis (CBA) was conducted in accordance with the guideline developed by the Harvard School of Public Health in collaboration with the Bill & Melinda Gates Foundation to monetize the potential costs and benefits of implementing VNA technology in the Brazilian Public Health System (SUS – *Sistema Único de Saúde*).^(5)^ These values were compared using the return on investment (ROI) measure, according to the formula which indicates the extent to which the benefits of implementing VNA outweigh its costs. As an *ex-ante* analysis, it is based on assumptions from the extant literature and expert opinion. All monetary values presented in this study were adjusted according to the Brazilian Consumer Price Index (IPCA *- Índice Nacional de Preços ao Consumidor Amplo*) as of March 2023. Where applicable, the cost and benefit flows were calculated as present values using a discount rate of 5% per year.^(6)^

### Analysis of VNA implementation in a diagnostic imaging center

The first analysis considers implementing VNA in a DIC. The costs considered include infrastructure and human resource expenses—such as computerization, internet access, digital certificates, and personnel—for implementing and maintaining the technology.

Cost estimates were divided into one-time and monthly costs. The former covers the initial investment required to adapt the DIC for VNA connection, including computerizing the DIC and acquiring a digital certificate to connect to the Brazilian National Health Data Network (RNDS - *Rede Nacional de Dados em Saúde*). The latter accounts for the regular maintenance of VNA and its ongoing integration with the RNDS. Being an *ex-ante* study, it was not feasible to provide a comprehensive account of the costs associated with the maintenance and updating of VNA systems and hardware. Consequently, proxies were employed to estimate the costs involved in these processes.

Table 1 depicts the reference values for calculating the costs of connecting the DIC to VNA. The cost of obtaining the digital certificate was estimated based on the value of a digital certificate from ICP-Brasil (*Infraestrutura de Chaves Públicas Brasileira*) as of March 2023. The cost of computerizing the DIC was estimated using data from the *Projeto Piloto de Apoio à Implementação da Informatização na Atenção Primária à Saúde.*^(7)^ The cost of maintaining computerization in the DIC was based on the *Programa de Apoio à Informatização e Qualificação dos Dados da Atenção Primária à Saúde (Informatiza APS).* In addition, monthly costs, including salary and benefits for a system analyst responsible for technology integration and technical support, were estimated using data from the *Relação Anual de Informações Sociais* (RAIS) of December 2021, assuming 200 working hours per month.

**Table 1:** Annual costs (R$) to connect the diagnostic imaging center to Vendor Neutral Archive

Item	Value (R$)
One-time costs	
Computerization	R$ 10.681,51
Digital certificate	R$ 500,00
Monthly costs	
Computerization (12 months)	R$ 25.635,63
System analyst (12 months)	R$ 154.883,88
Total	R$ 191.701,02

The benefits of implementing VNA at the DIC were divided into cost savings and environmental benefits. Cost savings arise from reducing the number of unnecessary exams and medical appointments, reducing the printing of—and need for personnel who print and deliver—exam results, and saving physical space for storing printed exams. Environmental benefits include lowering the DIC’s carbon footprint, expressed in tons of carbon dioxide equivalents (CO2e)—a metric that quantifies greenhouse gas emissions resulting from human activities and production^(8)^—and reducing waste volume by printing fewer exam results and reducing the number of unnecessary exams.

To calculate the cost reduction of implementing VNA in the DIC, 28% of the exams performed without VNA was assumed to be unnecessary.^(9)^ This percentage represents the expected reduction in unnecessary exams considered in the analysis. In addition, eliminating each unnecessary exam was assumed to also eliminate one corresponding medical appointment.

The reference value for the cost of a medical appointment was estimated at R$ 31,21. This estimate was based on the cost of 15 minutes of a family physician’s average salary in Brazil, assuming a work schedule of 200 hours per month, according to RAIS data from December 2021. While the RAIS database provides only the base gross salary received by physicians, our estimate incorporates the additional labor costs required by Brazilian labor law (*Consolidação das Leis do Trabalho* - CLT) for the employer, such as the 13^th^ salary (an additional monthly salary paid at the end of the year), one-third vacation bonus (an extra payment equivalent to one-third of the monthly salary paid during annual leave), *Fundo de Garantia do Tempo de Serviço* (FGTS) – a severance indemnity fund to which employers contribute 8% of the employee’s monthly salary), and the *Instituto Nacional do Seguro Social* (INSS) – the Brazilian social security system). These components were added to calculate the total employer cost, as detailed in table 2.

**Table 2:** Reference value (R$) used for a medical appointment based on the average salary of a family physician in Brazil

Description (family physician)	Value (R$)
Gross salary	R$ 17.556,71
Provision for 13th salary	R$ 1.463,06
Provision for one-third vacation bonus	R$ 487,69
FGTS	R$ 1.404,54
FGTS provision on 13th salary and vacations	R$ 156,06
INSS	R$ 3.511,34
INSS provision on 13th salary and vacations	R$ 390,15
Total employee cost	R$ 24.969,54
Value of 15 minutes of a medical appointment	R$ 31,21

Data extracted from the Relação Anual de Informações Sociais – RAIS. FGTS: *Fundo de Garantia do Tempo de Serviço*; INSS: *Instituto Nacional do Seguro Social.*

As depicted in table 3, the unit cost for each type of exam was obtained from the average outpatient transfer values reported in the *Sistema de Gerenciamento da Tabela de Procedimentos, Medicamentos e OPM do SUS* (SIGTAP) for January 2023, with the exception of those involving the use of contrast.

**Table 3:** Reference value (R$) used for each type of imaging exam

Imaging item	Value (R$)
Radiography	R$ 9,94
Ultrasonography	R$ 31,24
Computed tomography	R$ 104,23
Magnetic resonance imaging	R$ 275,36
Bone densitometry	R$ 55,10
Mammography	R$ 43,33
Echocardiography	R$ 132,62

Information from the *Sistema de Gerenciamento da Tabela de Procedimentos, Medicamentos e OPM do SUS* – SIGTAP (Available from: http://sigtap.datasus.gov.br/tabela-unificada/app/sec/inicio.jsp). Amounts for January 2023.

To estimate the cost reduction in printing, the average cost of four hospitals in Brazil’s Central-West and Southeast regions was used as a reference for each type of exam in 2023, as shown in table 4. For the CBA, an optimistic scenario was assumed in which all DICs printed 100% of the exams performed. With the implementation of VNA, DICs would no longer print the results of any exam.

**Table 4:** Printing costs (R$) per type of exam

Type of exam	Cost per print
Radiography	R$ 2,25
Ultrasonography	R$ 8,94
Computed tomography	R$ 3,01
Magnetic resonance imaging	R$ 6,02
Bone densitometry	R$ 5,96
Mammography	R$ 23,10
Echocardiography	R$ 7,45

Data sent by the diagnostic imaging centers analyzed.

The reduction in storage costs was calculated by assessing the physical space that would become unnecessary for storing imaging exams. This calculation was based on the following assumptions and proportions: (i) a room with a height of 2.5 meters; (ii) only 50% of the room’s height can be occupied with imaging exams per meter; (iii) for each square meter occupied by printed exams, an additional square meter must remain free. The calculation also considered the number of A3 and A4 sheets and radiological films required to print each type of exam, as shown in table 5, along with their respective dimensions. Moreover, a reference value of R$ 3.222,61 per square meter was used for monetization.^(10)^

**Table 5:** Number of sheets used for printing exams

	Number of A3 sheets per exam	Number of A4 sheets per exam	Number of X-ray films per exam
Radiography	1	1	0
Ultrasonography	0	6	0
Computed tomography	2	2	0
Magnetic resonance imaging	4	2	0
Bone densitometry	0	4	0
Mammography	0	2	6
Echocardiography	0	5	0

Data sent by the diagnostic imaging centers analyzed.

To calculate the reduction in the number of personnel responsible for printing and delivering imaging exams, the monthly salary of an administrative assistant working 220 hours per month was used as a reference, based on data from the RAIS for 2021. After incorporating labor charges and additional benefits, this was estimated at R$ 3.347,63 per administrative assistant.

Regarding environmental benefits, the savings from reduced solid waste collection were calculated based on the weight of exam results that were no longer printed. The reference value for one ton of waste was determined to be R$ 501,80, reflecting the average charge for municipal waste disposal in Brazil.^(11)^

For the amount of CO_2_e emitted per imaging examination unit, assumptions were made as follows: 0.8 kg for radiography, bone densitometry, and mammography;^(12)^0.65kg for ultrasonography and echocardiography;^(13)^ 9.2kg for computed tomography;^(12)^and 17.5 kg for magnetic resonance imaging.^(12)^Each ton of CO_2_e emitted was estimated at R$ 2,28.^(14)^

### Analysis of Vendor Neutral Archive implementation in SUS

The analysis for the SUS scenario covers the adoption of VNA in all DICs that performed at least one of the aforementioned imaging exams in 2022. It also includes all institutions whose outpatient procedures were funded by the SUS in 2022, as recorded in the *Sistema de Informação Ambulatorial do SUS (SIA-SUS)*, accessible through DATASUS.^(15)^

Outpatient SIA-SUS procedures were divided into subgroups and then aggregated by health institution using the *Cadastro Nacional de Estabelecimentos de Saúde* (CNES) code. Table 6 presents the total number of exams observed, categorized by the type of imaging exam.

**Table 6:** Distribution of imaging exams by type, considering the health institutions that performed at least one imaging exam for the SUS in 2022

Imaging item	Number of exams	%
Radiography	68,920,283	65.26
Ultrasonography	19,239,935	18.22
Computed tomography	8,918,130	8.44
Magnetic resonance Imaging	1,923,225	1.82
Bone densitometry	599,843	0.57
Mammography	4,249,479	4.02
Echocardiography	1,757,104	1.66
Total	105,607,999	100.00

Data extracted from SIA-SUS for 2022 (Available from: https://datasus.saude.gov.br/informacoes-de-saude-tabnet/).

In contrast to the DIC analysis, the SUS analysis incorporates all development costs associated with the Banco de Imagens project’s VNA, encompassing the entire technology development process from 2019 to 2023, including personnel costs (salaries and wages), server and cloud storage expenses, licenses, and other costs such as equipment, training, and external consulting. As of March 2023, VNA’s total development costs, adjusted for inflation, amounted to R$ 11.591,797,24.

## RESULTS

**Figure 1:**
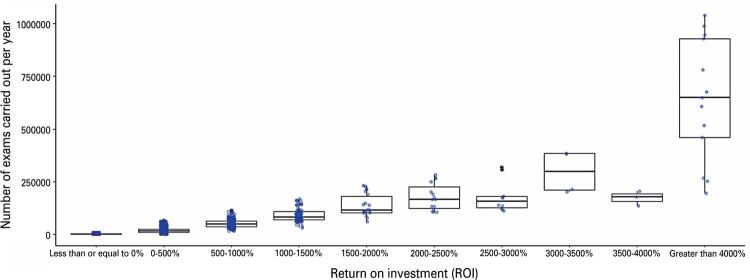
Range of return on investment for the number of exams performed per healthcare institution Data extracted from SIA-SUS for 2022 (Available from: https://datasus.saude.gov.br/informacoes-de-saude-tabnet/).

The first significant result, shown in figure 1, demonstrates a positive, albeit non-linear, relationship between the number of imaging exams produced and the financial return for the analyzed healthcare institution.

Table 7 reveals that approximately 77% of the institutions in the sample would experience a negative ROI in the first year of VNA implementation. This is attributed to the relatively small number of exams performed, with up to 50% of the sample exhibiting a negative ROI, conducting only 720 imaging exams in 2022. In addition, a lower threshold of approximately 11,500 exams exists, beyond which the ROI from implementing VNA is likely to become positive.

**Table 7:** Distribution of healthcare institutions and their descriptive statistics, according to return on investment ranges

Return on investment	Number of hospitals	Minimum number of exams performed per hospital	Median number of exams performed per hospital	Maximum number of exams performed per hospital
Less than 0%	10,002	1	720	11,423
Between 0 and 100%	1,304	2,047	11,832	22,815
Equal to or greater than 100%	1,729	4,135	27,464	1,037,875

Data extracted from SIA-SUS for 2022 (Available from: https://datasus.saude.gov.br/informacoes-de-saude-tabnet/).

The number of exams conducted by institutions varies greatly depending on their economic return range. For institutions with a negative return, the range of exams performed spans from 1 to 11,423. Meanwhile, for those with a return between 0 and 100%, this range extends from 2,047 to 22,815 exams. Lastly, for institutions with a return equal to or greater than 100%, the range extends from 4,135 to 1,037,875 exams. These overlapping ranges can be attributed to the positive, non-linear relationship between the number of exams produced and the return, as well as the disparity in production and printing costs among different exam types.

Institutions conducting more expensive exams require fewer exams to achieve non-negative returns. Conversely, those primarily performing radiographs, the least expensive of the considered types of imaging exams, may require a higher volume of exams to achieve similar returns.

Based on the results obtained from individual health institutions, the study aggregates them at the SUS level. Table 8 presents the results of the CBA considering different time horizons. The first year of analysis includes not only the costs of developing, implementing, and maintaining VNA at the DIC but also the cost savings and environmental benefits for the DIC. Subsequent years only consider the variable costs associated with implementing and maintaining VNA.

**Table 8:** Return on the added value of one real (R$) invested in implementing Vendor Neutral Archive within the SUS, expressed in present value using a discount rate of 5% per year

Time horizon	Cost (million R$)	Cost reduction (million R$)	Environmental benefits (million R$)	Return per real invested (R$)	Return on investment %
1	R$ 2.510,42	R$ 2.649,08	R$ 2,70	R$ 0,0563	5.63
2	R$ 4.751,44	R$ 5.172,02	R$ 5,29	R$ 0,0896	8.96
3	R$ 6,885.75	R$ 7.574,81	R$ 7,74	R$ 0,1012	10.12

The estimated ROI for the first year after implementing VNA in the SUS, under the study scenario, is 5.63%, corresponding to a return of R$ 0,0563 per real invested. This return increases with longer time horizons considered in the analysis. After three years of implementation, the estimated ROI would rise to 10.12%, equivalent to a return of R$ 0,1012 per real invested.

### Sensitivity analysis

The analyses rely on two sources of uncertainty: simulator assumptions and the sample of healthcare institutions performing imaging exams for the SUS. To provide information on the magnitude of these uncertainties, deterministic and probabilistic sensitivity analyses of the results were conducted, considering a one-year time horizon.

**Figure 2:**
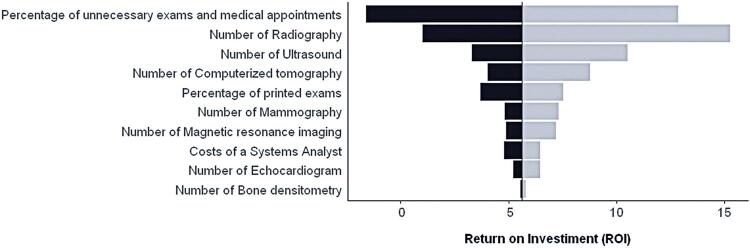
Tornado diagram of the deterministic sensitivity analysis The black and gray bars represent the effects of a 10% reduction and 10% increase in the variable of interest, respectively.

Figure 2 displays the tornado diagram, illustrating the change in return per real invested owing to variations of around 10% in some parameters. The variables with the most substantial impact on the calculated return were the percentage of unnecessary exams and appointments and the total number of radiography and ultrasonography exams. These two variables accounted for 65.26% and 18.22% of all imaging exams in the sample, respectively (as indicated in Table 6). The inclusion of 28% of unnecessary exams and appointments in the calculation of the cost reduction associated with implementing VNA is depicted in the diagram as a pivotal parameter influencing the economic return of VNA for the SUS.

**Figure 3:**
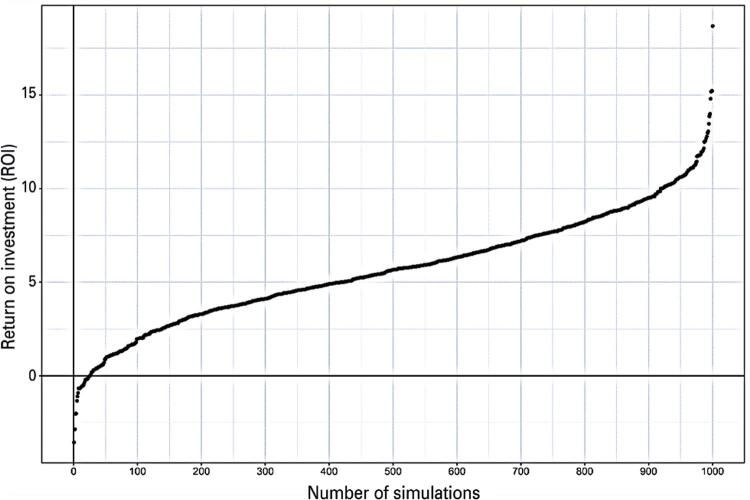
Acceptability curve of the cost-benefit analysis in the context of the SUS

Figure 3 depicts the acceptability curve, indicating the cumulative number of simulations that reached a certain level of ROI in adopting VNA in the SUS. The sample’s uncertainty of the CBA results in an average ROI of 5.67%, ranging from -3.56% to 18.69%, with a standard deviation of 2.96% for a one-year time horizon. Moreover, the ROI is positive in 97.5% of the 1,000 simulations conducted. These findings suggest that the ROI is generally positive. However, the standard deviation is relatively high compared to the mean estimate, as is the range between the minimum and maximum values. This dispersion of data underscores the importance of interpreting the study results with caution.

## DISCUSSION

Developing and implementing cloud-based technology to store medical imaging exams and reports requires significant investment, especially in the design phase. However, adopting this approach can yield substantial benefits for patients, healthcare professionals, and healthcare institutions.

A positive short-term financial return was observed in the SUS, with a non-linear relationship between the number of imaging exams conducted and the financial return. Considering a scenario in which only establishments that conduct more than 11,500 exams per year are included—according to the previously identified threshold, which suggests that a positive return is likely beyond this volume—the estimated ROI would be 347.22% in the first year following implementation, corresponding to a return of R$ 3,4722 for every real invested. These findings indicate that the financial benefit of implementing the VNA in the SUS depends on its scalability across the healthcare system, suggesting that a phased implementation may be an effective strategy to integrate this system. As the number of healthcare institutions adopting the VNA increases, so does the ROI for the healthcare system.

The sensitivity analysis reveals that the financial return of VNA is most influenced by two factors: (i) the percentage of unnecessary exams and appointments and (ii) the proportion of radiographs to the total number of imaging exams performed in the DIC. The probabilistic sensitivity analysis indicates that uncertainties arising from the sample do not significantly affect the estimates of the financial return from implementing VNA.

However, this analysis has its limitations. Because no estimates are available for the cost of potential repairs or server capacity upgrades for VNA, costs may be underestimated over longer periods of time. In addition, the results are highly sensitive to the percentage of unnecessary exams and medical consultations, making this percentage a significant factor in the financial return. Estimates of this percentage were only found in the international literature, so obtaining an estimate of this percentage in Brazil is advisable.

The results suggest that the financial return of implementing VNA in the SUS is influenced by the sample composition, which primarily comprises small hospitals. Typically, implementations in large hospitals conducting numerous imaging exams tend to yield higher financial returns because of the greater volume of tests performed. However, it is noteworthy that in practice, small hospitals can seamlessly aggregate, leveraging shared resources and infrastructure, which can lead to a positive impact similar to a larger hospital. This aggregation enables small hospitals to collectively achieve economies of scale and efficiency gains, thereby enhancing the overall financial return of VNA implementation within the SUS.

The analysis did not consider secondary benefits that could positively impact patient health, such as expedited and enhanced patient care, optimized physician schedules, smaller waiting lines, long-term patient monitoring, digitalization of diagnostic centers, and support for AI tools. Many of these benefits—including improvements in patient care and the broader positive effects of using AI technologies in medical practices—are challenging to quantify in monetary value. Thus, the benefits for the SUS and society, in terms of efficiency and equity, may exceed those observed in this study.

To summarize, this study evaluated the potential financial benefits of implementing VNA for medical image storage within the SUS, aiming to provide decision-making insights to managers using similar technologies. Notably, this is an *ex-ante* study based on assumptions. Therefore, although the results offer valuable guidance, they require cautious interpretation under specific conditions.

## CONCLUSION

This study underscores the strategic value of Vendor Neutral Archive for cloud-based medical image storage in the Brazilian Public Health System context despite initial challenges related to investment and methodological limitations. The scalability of Vendor Neutral Archive implementation provides evidence of positive short-term financial returns, indicating its capacity to enhance healthcare efficiency and sustainability. However, for informed decision-making, managers must pursue more precise estimates tailored to the Brazilian reality, considering the often-overlooked secondary benefits associated with Vendor Neutral Archive adoption.

Moreover, the findings suggest that while the initial investment in Vendor Neutral Archive technology across the Brazilian Public Health System is substantial, the return can be realized in less than one year. This rapid return period highlights the potential of Vendor Neutral Archive to quickly deliver financial benefits and optimize resource allocation. By addressing these crucial aspects, managers can leverage Vendor Neutral Archives innovative potential to optimize resource management within the Brazilian healthcare system.
